# Feasibility of treatment discontinuation in chronic myeloid leukemia in clinical practice: results from a nationwide series of 236 patients

**DOI:** 10.1038/s41408-018-0125-0

**Published:** 2018-12-02

**Authors:** Juan Carlos Hernández-Boluda, Arturo Pereira, Irene Pastor-Galán, Alberto Alvarez-Larrán, Alisa Savchuk, José Manuel Puerta, José María Sánchez-Pina, Rosa Collado, Alvaro Díaz-González, Anna Angona, Miguel Sagüés, Valentín García-Gutiérrez, Concepción Boqué, Santiago Osorio, Rolando Vallansot, Luis Palomera, Arantxa Mendizábal, Luis Felipe Casado, Manuel Pérez-Encinas, Raúl Pérez-López, Francisca Ferrer-Marín, Fermín Sánchez-Guijo, Carmen García, Natalia de las Heras, José Luis López-Lorenzo, Francisco Cervantes, Juan Luis Steegmann, on behalf of the Grupo Español de Leucemia Mieloide Crónica (GELMC)

**Affiliations:** 1 Hematology Department, Hospital Clínico Universitario-INCLIVA, Valencia, Spain; 2 Hemotherapy and Hemostasis Department, Hospital Clínic-IDIBAPS, Barcelona, Spain; 3 Hematology Department, Hospital Clínic-IDIBAPS, University of Barcelona, Barcelona, Spain; 4 Hematology Department, Hospital Universitario La Princesa, Madrid, Spain; 5 Hematology Department, Hospital Universitario Virgen de las Nieves, Granada, Spain; 6 Hematology Department, Hospital 12 de Octubre, Madrid, Spain; 7 Hematology Department, Hospital General Universitario, Valencia, Spain; 8 Hematology Department, Hospital La Fe, IIS La Fe, Valencia, Spain; 9 Hematology Department, Hospital del Mar-IMIM, Barcelona, Spain; 10 Hematology Department, Hospital Universitario de Girona, Doctor Josep Trueta, Girona, Spain; 11 Hematology Department, Hospital Ramón y Cajal-IRYCIS, Madrid, Spain; 12 Hematology Department, Institut Català d’Oncologia, Hospital Duran i Reynals, Hospitalet de Llobregat, Spain; 13 Hematology Department, Hospital General Universitario Gregorio Marañón, Madrid, Spain; 14 Hematology Department, Hospital Universitario Joan XXIII, Tarragona, Spain; 15 Hematology Department, Hospital Clínico Universitario Lozano Blesa, IIS Aragón, Zaragoza, Spain; 16 Hematology Department, Hospital Universitario de Álava, Vitoria, Spain; 17 Hematology Department, Hospital Virgen de la Salud, Toledo, Spain; 18 Hematology Department, Hospital Clínico Universitario, Santiago de Compostela, Spain; 19 Hematology Department, Hospital Clínico Universitario Virgen de la Arrixaca, Murcia, Spain; 20 Hematology and Medical Oncology Department, Hospital Morales Meseguer-CIBERER, IMIB-Arrixaca, UCAM, Murcia, Spain; 21 Hematology Department, Hospital Universitario de Salamanca, IBSAL, Salamanca, Spain; 22 Hematology Department, Hospital General Universitario de Alicante, Alicante, Spain; 23 Hematology Department, Complejo Asistencial Universitario de León, León, Spain; 24 Hematology Department, Fundación Jiménez Díaz, Madrid, Spain

## Abstract

Over half of chronic myeloid leukemia (CML) patients in deep molecular response do not lose the major molecular response (MMR) after stopping treatment with tyrosine kinase inhibitors (TKI). This strategy is safe in clinical trials, but its applicability in the real-life setting remains unsettled. We describe the outcomes after TKI discontinuation in a nationwide series of 236 CML patients. Median follow-up from treatment discontinuation was 21.5 months and 5 patients died from CML-unrelated causes. TKI therapy was reinitiated due to MMR loss (*n* = 52), increase ≥ 1 log in *BCR-ABL* transcript level without losing MMR (*n* = 12), patient preference (*n* = 2), and withdrawal syndrome (*n* = 1). Treatment-free remission rate at 4 years was 64% (95% confidence interval, CI: 55%–72%). Cumulative incidence of molecular recurrence at 3 years was 33% (95% CI: 26%–38%). TKI treatment for < 5 years and MR4.5 duration shorter than 4 years were both associated with higher incidence of molecular recurrence. No patient had disease progression. Response status at last control was: MR4.5 (*n* = 196), MR4 (*n* = 15), MMR (*n* = 14), complete cytogenetic response (*n* = 10), and other (*n* = 1). A significant increase in Hb and cholesterol levels was observed after imatinib withdrawal. Our results demonstrate that TKI treatment discontinuation is feasible in real-life clinical practice.

## Introduction

Life expectancy of patients with chronic myeloid leukemia (CML) is currently approaching that of the general population, as a result of the successful treatment with tyrosine kinase inhibitors (TKIs)^1^. During the first 1 or 2 years of treatment, most patients achieve a significant decrease in the leukemic burden to a level that protects them from disease transformation^2^. Later on, about 30–50% of patients achieve a state of undetectable or nearly undetectable molecular residual disease that is generally sustained if treatment compliance is good^3–5^. Despite such excellent efficacy, several studies have demonstrated the persistence of leukemic stem cells in CML patients with deep molecular response (DMR)^6,7^, with this supporting the need to maintain treatment indefinitely to prevent disease recurrence. However, mere persistence of leukemia stem cells at such low level may not predict disease progression or resistance. Of concern, long-term TKI therapy is not devoid of potential medical risks for the patients^8^ and poses a considerable economic burden to the healthcare systems^9^, although this has been eased with the arrival of generic imatinib.

The notion of the need of lifelong treatment in CML has been challenged in recent years by the results of numerous clinical trials demonstrating that over half of the patients in DMR can remain relapse-free after stopping TKI therapy^10–13^. Data on more than 2500 patients who discontinued treatment in sustained DMR have demonstrated that this approach is feasible in the setting of controlled clinical trials ^14–16^. Most relapses are observed during the first year after treatment cessation but late relapses can occasionally occur, and therefore close monitoring is of crucial importance for the safety of this strategy^17^.

It is of note that achieving a treatment-free remission (TFR) has quickly become a key goal of CML therapy^18,19^, even though no general consensus on its applicability in clinical practice has been established^14,15,20^. Moreover, information on the safety of TKI cessation outside of clinical trials is still limited^21–24^. Only recently, a number of publications have focused on the selection of optimal candidates for treatment cessation outside of clinical trials and on the minimum requirements for an adequate monitoring^15,16,18,25–27^. In this study, we describe the outcomes after TKI discontinuation in a large series of CML patients from Spain.

## Methods

### Patients

The present retrospective study comprised a series of 236 patients in chronic-phase CML, who discontinued TKI treatment outside of clinical trials between April 2009 and February 2018 in 33 Spanish institutions associated to the Grupo Español de Leucemia Mieloide Crónica (GELMC). After central review, all cases met the following inclusion criteria: (a) TKI treatment duration for at least 3 years; (b) sustained MR4.5 (*BCR-ABL1* International Scale (IS) ≤ 0.0032% or undetectable *BCR-ABL1* in samples with ≥ 32,000 *ABL1* transcripts) in ≥ 4 consecutive determinations (one single point in MR4 was acceptable) during a minimum of 2 years before treatment discontinuation; (c) strict molecular monitoring in a reference laboratory expressing the results on the IS with sensitivity > 4-log. Patients who had undergone allogeneic hematopoietic stem cell transplantation were excluded, but other previous pharmacological therapies were permitted.

Molecular relapse was defined as consecutively detectable peripheral blood *BCR-ABL1* transcripts showing a ≥ 1 log increase or loss of major molecular response (MMR) in any single sample. During the study period, both situations could trigger TKI resumption at the discretion of the attending physician based on the prevailing recommendations at that time.

The study was approved by the Ethics Committee of the Hospital Clínico Universitario of Valencia and registered by the AEMPS (Agencia Española de Medicamentos y Productos Sanitarios), under the reference INC-IMA-2017–01. Informed consent for the inclusion in the study was obtained in accordance with the requirements by local ethics committees.

### Statistical analysis

Quantitative data are expressed as median and interquartile range (IQR) and qualitative data as percentages. The non-parametric Skillings–Mack test was used for comparing the laboratory values at baseline and at different time points after treatment discontinuation^28^. Differences in the distribution of categorical and continuous variables were evaluated by the *χ*^2^-test and the Mann–Whitney *U*-test, respectively.

TFR was estimated by the method of Kaplan–Meier and defined as the time from TKI discontinuation to the date of restarting therapy for any reason or the date of last contact if treatment was not restarted. Incidence of molecular relapse was calculated using the cumulative incidence function with resumption of TKI treatment in the absence of molecular relapse and death in MMR as competing events. For the remaining patients, follow-up was censored at the date of the last molecular monitoring. The following patients’ clinical characteristics were evaluated for their potential relationship with the incidence of molecular relapse: sex, age at discontinuation, Sokal risk score, type of *BCR-ABL1* transcript (e13a2 vs. e14a2), history of TKI resistance, number of TKI lines before discontinuation (1 vs. more than one), type of TKI at the time of discontinuation (imatinib vs. others), prior exposure to interferon, aggregated time on TKI treatment, and duration of MR4.5 before discontinuation. Optimal prognostic cutoffs for time on TKI treatment and duration of MR4.5 before discontinuation were selected by receiver-operating characteristic curve analysis of molecular relapse at 1 year. Univariate analyses of factors predicting molecular relapse were done within the framework of competing risks by the method of Fine and Gray^29^.

All thee performed with IBM SPSS 22.0 (SPSS, Chicago, IL, USA) and Stata 11 (https://www.stata.com).

## Results

### Patients’ characteristics and clinical outcome

Demographics and treatment history of the whole series is summarized in Table 1. Median age at time of stopping TKI treatment was 61 years (IQR: 52–72) and 123 (52%) patients were females. Sokal risk score at diagnosis was high in only 17 (8%) patients. *BCR-ABL* transcript was typical in 232 cases (e13a2 = 70, e14a2 = 104, both = 6, p210 but undetermined isoform = 52) and atypical in 4 cases (e14a3 = 1, e13a3 = 1, e1a2 = 2). Frontline TKI treatment consisted of imatinib (*n* = 217), nilotinib (*n* = 15), dasatinib (*n* = 2), or bosutinib (*n* = 2). Second-line TKI treatment consisted of imatinib (*n* = 3), nilotinib (*n* = 22), or dasatinib (*n* = 27). Third-line TKI treatment was imatinib (*n* = 3), nilotinib (*n* = 11), bosutinib (*n* = 4), or ponatinib (*n* = 2). At the time of stopping treatment, most patients (74%) were receiving imatinib. All patients were in DMR, but the main reasons for TKI cessation were the presence of side effects or a concomitant disease (*n* = 66), an attempt to achieve a TFR in the absence of clinically relevant TKI toxicity (*n* = 166) and pregnancy or planning pregnancy (*n* = 4).

**Table 1:** Demographics and treatment history of 236 chronic-phase CML patients who discontinued TKI treatment in DMR in Spain from April 2009 to February 2018

Age at diagnosis, year^a^	50 (40–61)
Age at TKI discontinuation, year^a^	61 (52–72)
Sex, females (%)	123 (52)
Sokal risk score, *n* (%)	
Low	129 (60)
Intermediate	69 (32)
High	17 (8)
Unknown	21
Time from diagnosis to TKI discontinuation, months^a^	130 (96–162)
Prior interferon treatment, *n* (%)	55 (23)
TKI lines before TKI discontinuation, *n* (%)	
One	184 (78)
Two	32 (14)
Three	20 (8)
TKI at the time of treatment cessation, *n* (%)	
Imatinib	175 (74)
Nilotinib	41 (17.5)
Dasatinib	17 (7)
Bosutinib	1 (0.5)
Ponatinib	2 (1)
History of resistance to any TKI, *n* (%)	17 (7)
Duration of TKI treatment, months^a^	123.5 (93–150)
Time in MR4.5 before TKI discontinuation, months^a^	68 (40–100)

*DMR* deep molecular response, *TKI* tyrosine kinase inhibitor

^a^Median (interquartile range)

**Fig. 1:**
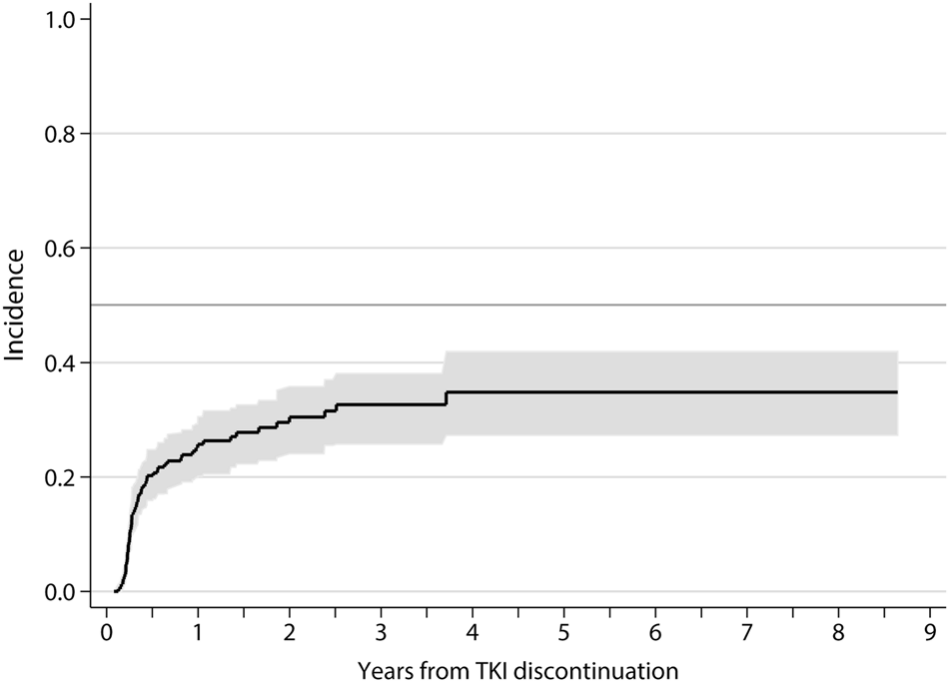
Cumulative incidence (± 95% CI) of molecular relapse after TKI discontinuation

**Fig. 2:**
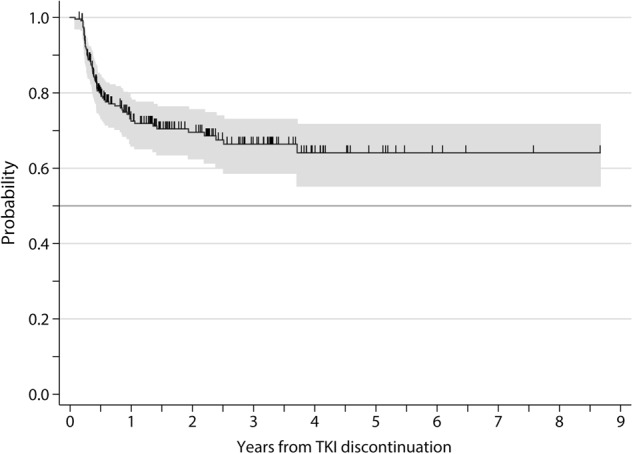
Treatment-free remission (± 95% CI) after TKI discontinuation

At the study closing date in 15 May 2018, median follow-up from treatment discontinuation was 21.5 months (IQR: 10–39) and 5 patients had died while in MMR due to CML-unrelated causes. In 67 patients (28%), TKI therapy was reinitiated due to molecular relapse (loss of MMR: *n* = 52, increase ≥ 1 log in *BCR-ABL* transcript level at two consecutive assessments without losing MMR: *n* = 12), patient preference (*n* = 2), and severe withdrawal syndrome (*n* = 1). One additional patient lost MMR after 20 months from treatment cessation but decided not to be retreated, with spontaneous recovery of MMR in subsequent determinations. The cumulative incidence of molecular recurrence was 20% (95% confidence interval (CI): 16%–25%) at 6 months, 26% (95% CI: 21%–30%) at 1 year, and 33% (95% CI: 26%–38%) at 3 years (Fig. 1). Forty-nine relapses (75% of total) occurred in the first 6 months, eight between months 7 and 12, and eight later than 12 months. The latest loss of MMR was detected 30 months after TKI discontinuation. One patient restarted treatment 44 months after TKI discontinuation due to ≥ 1 log increase in *BCR-ABL1* transcripts in two consecutive positive samples without losing MMR. The probability of TFR at 1 and 4 years was 72.5% (95% CI: 66%–78%) and 64% (95% CI: 55%–72%), respectively (Fig. 2). Among the 164 patients who remained in MMR without treatment, 10 lost MR4.5 (without loss of MR4) and 19 lost MR4 on at least one occasion during the follow-up period. The aggregated time off treatment for the whole series was 4559 months.

### Safety and laboratory data after TKI discontinuation

All patients remained in complete hematologic response and none progressed to the advanced phases of CML. At the time of restarting treatment, the median *BCR-ABL1* IS was 0.3% (IQR: 0.1–1.17), with this value being > 5% in only seven instances. Most patients (81%) received the same TKI that they were taking before treatment cessation. In 44 of the 52 patients (85%) who lost MMR, treatment was initiated within the first month after detection of the molecular relapse. Median follow-up after treatment resumption was 20 months (IQR: 9–37). Among the 64 patients who restarted treatment due to molecular recurrence, 46 of 52 regained MMR after a median time of 3 months (IQR: 1–5), 50 of 64 regained MR4 after a median time of 3.5 months (IQR: 2–6), and 47 of 64 regained MR4.5 after a median time of 5 months (IQR: 2–8). Six patients did not regain MMR, but the follow-up of five of them was still short (< 3 months). The remaining one was a heart transplantation recipient with chronic renal failure, who was unable to maintain TKI therapy continuously, due to several infectious episodes and pleural effusion. At last control, the response status was as follows: MR4.5 (*n* = 196), MR4 (*n* = 15), MMR (*n* = 14), complete cytogenetic response (*n* = 10), and other (*n* = 1).

A total of 51 patients (22%) developed musculoskeletal or joint pain after treatment cessation. In one patient, imatinib had to be reinitiated at low doses due to severe pain and joint deformities in both hands that did not respond to anti-inflammatory drugs.

In 66 patients in DMR, the main reason for TKI cessation was the presence of side effects or a concomitant disease. Among the 62 cases in whom evolutive data were available, a complete resolution of such toxicity was noted in 33 (53%) patients, a partial improvement was seen in 17 (28%) patients, whereas no significant change was observed in the remainder.

**Fig. 3:**
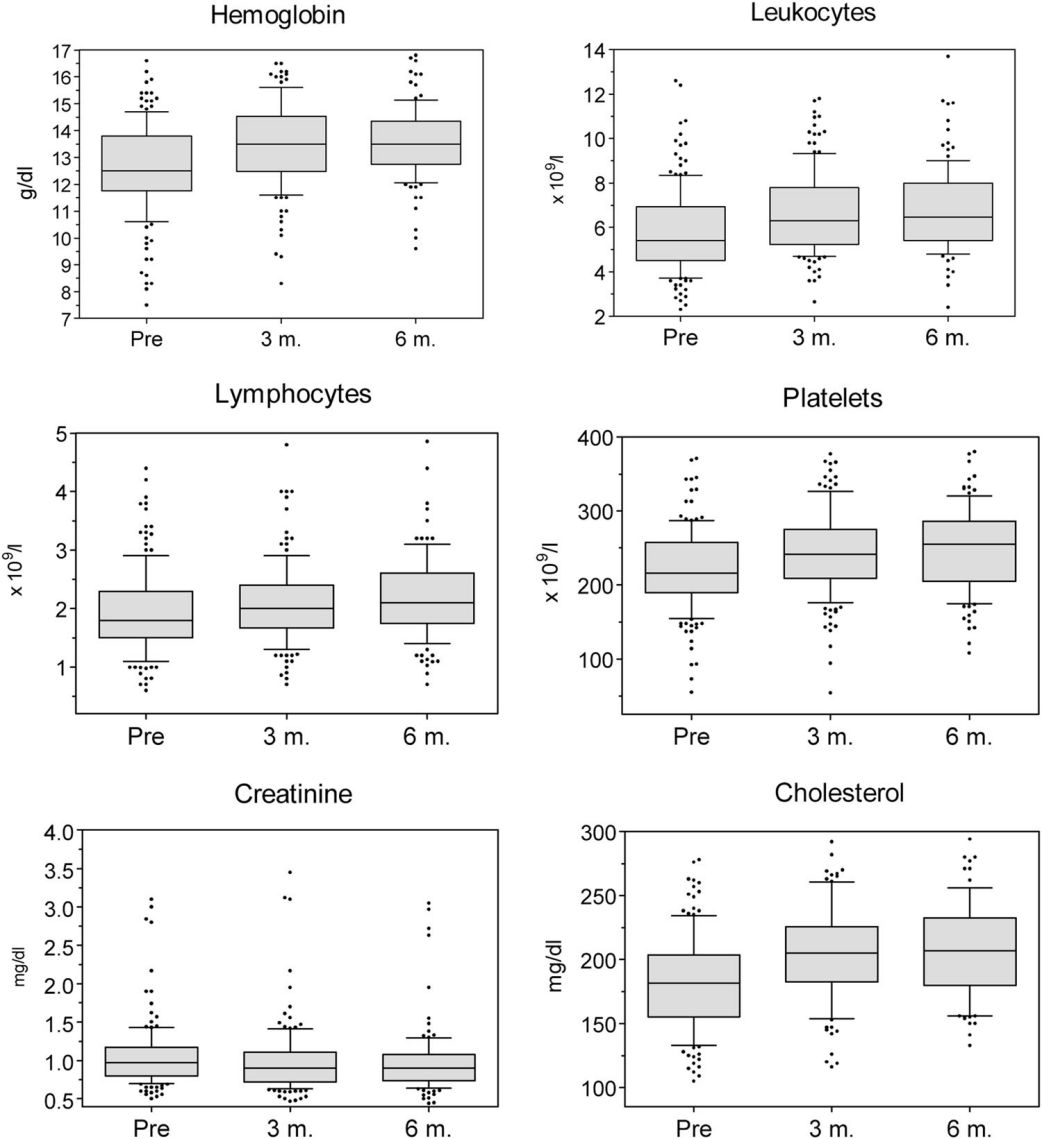
Evolution of selected analytical parameters during the first 6 months after imatinib discontinuation. *Changes in all the parameters were statistically significant (*P* < 0.001) by the Skillings–Mack test

We evaluated several hematological and biochemical parameters at baseline and at 3 and 6 months following TKI cessation. At the time of treatment discontinuation, patients on imatinib had on average lower values of Hb, leukocytes, and cholesterol, and higher creatinine levels than patients on nilotinib, although mostly within normal ranges (Table 2). In patients stopping imatinib, a significant increase in the Hb levels, leukocyte counts, total lymphocyte counts, and platelet counts was observed (Fig. 3). At 6 months, increases in Hb levels ≥ 1 g/dL and ≥ 2 g/dL were observed in 51% and 25% of patients, respectively. Among the anemic patients, the median Hb increase was 1.8 mg/dL (IQR: 0.9–2.5), with 72% and 47% of cases experiencing increases in Hb levels ≥ 1 g/dL and ≥ 2 g/dL, respectively. The serum creatinine decreased slightly, whereas cholesterol levels showed a significant increase (Fig. 3). Median increase in total cholesterol level at 6 months was of 29.5 mg/dL (IQR: 12.2–41.7), with 53% of patients with a baseline cholesterol < 200 mg/dL reaching a cholesterol level above such figure. By contrast, discontinuation of nilotinib was not followed by any relevant change in laboratory values, except for a mild decrease in the leukocyte counts (data not shown). Finally, the number of patients who discontinued other TKI was too small to draw any conclusions on this subject.

**Table 2:** Evolution of the laboratory values after stopping imatinib or nilotinib

At baseline				At 6 months after discontinuation		
	Imatinib	Nilotinib	*P*	Imatinib	Nilotinib	*P*
Hb, g/dL^a^	12.5 (11.7–13.7)	13.5 (12.5–14.8)	**0.002**	13.5 (12.7–14.3)	13.6 (12.4–14.8)	0.74
Leukocytes, × 10^9^/L^a^	5.4 (4.5.6.9)	7.8 (6.1–9.1)	**<** **0.001**	6.5 (5.4–8)	7.25 (6.1–9.1)	0.11
Lymphocytes, × 10^9^/L^a^	1.8 (1.5–2.3)	2 (1.6–2.4)	0.063	2.1 (1.75–2.6)	1.85 (1.6–2.5)	0.18
Platelets, × 10^9^/L^a^	215 (190–257)	223 (180–269)	0.59	255 (205–286)	214 (181–271)	0.09
Creatinine, mg/dL^a^	0.97 (0.8–1.17)	0.86 (0.72–1.05)	**0.025**	0.9 (0.73–1.08)	0.89 (0.72–1.05)	0.67
Total cholesterol, mg/dL^a^	181 (155–203)	206 (160–238)	**0.025**	207 (178–232)	172 (140–210)	**0.004**

^a^Median (interquartile range)

The Mann–Whitney *U*-test was used for comparisons

Numbers in bold are those with a significant *P*-value on the statistical analysis (*P* < 0.05)

### Predictors of molecular relapse

Table 3 summarizes the analysis of factors associated with increased risk of molecular relapse. As can be seen, shorter periods of both TKI treatment and RM4.5 before TKI discontinuation were significantly associated with increased risk of molecular recurrence. Indeed, the cumulative incidence of relapse at 3 years in patients who had received TKI treatment for more than 5 years was 30% (95% CI: 24%–38%), as compared with 59% (95% CI: 38%–80%) for those treated for a shorter period (Supplemental Figure S1). Patients in sustained MR4.5 for more than 4 years before TKI cessation had a 25% (95% CI: 18%–35%) cumulative incidence of molecular relapse at 3 years, as compared with 46% (95% CI: 34%–59%) for those with a shorter period in MR4.5 (Supplemental Figure S2). Type of TKI at the time of discontinuation (imatinib vs. others) had no effect on the subsequent risk of molecular relapse (subhazard ratio (SHR): 0.77; 95% CI: 0.44–1.33; *P* = 0.35).

**Table 3:** Factors potentially associated with an increased risk of molecular relapse after discontinuation of TKI treatment in CML

Covariate	No. patients per group	Subhazard ratio (95% CI)	*P-*value
Sex (male vs. female)	113 / 123	1.05 (0.64–1.71)	0.83
Age (> 60 vs. ≤ 60 years)	125 / 111	1.03 (0.63–1.69)	0.87
Sokal risk index (high vs. low-intermediate)	17 / 198	1.54 (0.73–3.25)	0.25
*BCR-ABL* transcript type (e13a2 vs. e14a2)	70 / 104	0.78 (0.43–1.42)	0.43
History of TKI resistance (yes vs. no)	17 / 219	1.14 (0.43–3.06)	0.28
No. of TKI lines before discontinuation (> 1 vs. 1)	52 / 184	1.21 (0.67–2.2)	0.51
TKI at the time of discontinuation (imatinib vs. others)	175 / 61	0.77 (0.44–1.33)	0.35
Prior exposure to interferon (yes vs. no)	55 / 181	0.53 (0.28–1.01)	0.055
Duration of TKI treatment (< 5 years vs. ≥ 5 years)	19 /217	2.59 (1.33–5.01)	**0.005**
Time in MR4.5 before discontinuation (< 4 years vs. ≥ 4 years)	82 / 153	2.06 (1.27–3.35)	**0.003**

*CI* confidence interval, *CML* chronic myeloid leukemia, *TKI* tyrosine kinase inhibitor

Numbers in bold are those with a significant *P*-value on the statistical analysis (*P* < 0.05)

According to Hughes and Ross^15^, optimal candidates for treatment discontinuation in clinical practice would be those fulfilling all the following criteria: (a) patients in chronic-phase CML, who have a low or intermediate Sokal risk score and a typical *BCR-ABL* transcript (e13a2 or e14a2); (b) at least 8 years on TKI treatment without history of resistance; and (c) sustained MR4.5 lasting ≥ 2 years. In the present series, 58% of patients fulfilled these criteria as optimal candidates for TKI discontinuation. However, the cumulative incidence of molecular relapse for the optimal candidates did not significantly differ from the “less than optimal” group (SHR: 0.85, 95% CI: 0.47–1.52, *P* = 0.58; Supplemental Figure S3).

## Discussion

In the present study, the safety of TKI discontinuation in clinical practice was evaluated in a nationwide series of 236 CML patients from 33 Spanish centers. Overall, the treatment-free survival was of 64% at 4 years. Most patients who fail to maintain TFR regained a DMR after 3–5 months of restarting treatment. No case of disease progression was documented. These data confirm the applicability of this treatment strategy outside of clinical trials in Spain.

In fact, the results from the current series compare favorably with the ~50% TFR rate reported in most clinical trials on TKI discontinuation^10–13^. Moreover, in 18% of our patients the reason for treatment resumption was an increase in *BCR-ABL1* transcript levels without losing MMR. It is currently known that a proportion of such cases, particularly those presenting an increase in *BCR-ABL1* transcript levels after the first 6 months of stopping treatment (i.e., “the late molecular relapses”)^12,17^ could have spontaneously regained DMR without therapy. However, this information was not available at the time of TKI cessation in our earlier cases, whereas in other instances the decision of resuming treatment before MMR loss might reflect the cautious approach of the attending physicians to prevent any complication related to stopping TKI therapy outside of a clinical trial. Nevertheless, the favorable TFR of the current series likely derives from the prolonged exposure to TKI treatment (median ~10 years) and the sustained duration in DMR (median ~5 years) before TKI discontinuation in our patients, which largely exceeded the minimal thresholds of clinical trials. Actually, these two variables may be the most important factors affecting the probability of molecular relapse-free survival^27^. In the recently published EURO-SKI trial^13^, the probability of maintaining MMR at 6 months was of 61% in imatinib-treated patients with DMR duration of more than 3.1 years, whereas it was of 44% only in those with a shorter period in DMR.

Strict molecular monitoring and a swift resumption of TKI treatment upon detection of molecular relapse are essential for the safety of the discontinuation strategy in clinical practice^26^. To this respect, Spain has an extensive network of certified laboratories for *BCR-ABL1* molecular monitoring in the public health system, where the great majority of CML patients are managed. In our series, most patients resumed TKI treatment within the first month after detection of MMR loss. Occasionally, treatment initiation was delayed due to an ongoing pregnancy. Of note, the *BCR-ABL1* transcript levels at the time of restarting treatment were above the threshold of 5% IS in only 7 cases, with this suggesting that an adequate monitoring was performed in the setting of common clinical practice. Indeed, disease resistance was not an issue in patients who required TKI therapy due to molecular relapse, in line with published data from clinical trials^14^.

Several findings on the evolution of the laboratory parameters after TKI discontinuation deserve to be mentioned. At the time of treatment cessation, the Hb levels and the leukocyte counts were significantly lower in patients receiving imatinib than in those on nilotinib treatment, with this suggesting a stronger myelosuppressive effect of the former. In fact, a dose-dependent inhibitory effect of imatinib on normal progenitor cells has experimentally been demonstrated in vitro^30^ and in vivo^31^. In a previous study including CML patients who were in DMR with imatinib 400 mg daily, a dose reduction to 300 mg daily resulted in a median Hb increase of 1 g/dL among the anemic patients^32^. Our present data confirmed that the myelosuppressive effect of long-term treatment with imatinib is reversible, as patients stopping this drug experienced a significant increase in all hematologic values^33^. In particular, the increase in Hb levels observed in anemic patients at 6 months from imatinib discontinuation (≥ 1 g/dL and ≥ 2 g/dL in 72% and 47% of patients, respectively) might be of clinical significance, as chronic fatigue has been identified as the most important factor limiting health-related quality of life in patients on long-term therapy with imatinib^34^. By contrast, nilotinib withdrawal did not appear to have any major impact on the hematologic values. With regard to the biochemical parameters, patients who stopped imatinib had higher serum creatinine levels at baseline, but experienced a significant reduction in creatinine levels afterwards, in line with previous reports suggesting that imatinib can decrease glomerular filtration^35,36^. On the other hand, it is well recognized the early onset hypercholesterolemia induced by nilotinib^37^, with such effect being potentially involved in the pathogenesis of the occlusive arterial events associated with this drug^38^. Current guidelines recommend a proactive approach towards controlling the hyperlipidemia induced by nilotinib with lifestyle interventions and lipid-lowering therapies^8^. Despite such recommendations, total cholesterol levels at the time of TKI discontinuation were significantly higher in nilotinib-treated patients than in those receiving imatinib (median difference ~20 mg/dL). Such situation changed completely after TKI treatment discontinuation, as cholesterol levels significantly increased following imatinib withdrawal. As a result of this tendency, at 6 months after treatment discontinuation patients who stopped imatinib ended up with total cholesterol levels that were significantly higher than those of patients stopping nilotinib. In this sense, plasma lipid levels have been reported to normalize within one month of imatinib treatment in small case series^39^. Imatinib has also been shown to delay the development of atherosclerosis in experimental models^40^ and to induce the regression of type 2 diabetes mellitus^41^. Consequently, the potential influence of cessation of imatinib treatment in the long-term risk of cardiovascular events needs to be evaluated in future studies.

Several experts’ recommendations^14–16,25,26^ and formal guidelines^18,27^ have recently been published focusing on the selection of candidates for TKI discontinuation in clinical practice. In general, there is consensus on the need to define a minimum duration of TKI exposure and DMR before treatment discontinuation is to be attempted. However, significant differences in the thresholds for the minimal duration of TKI exposure have been proposed, ranging from 3 to 8 years. In our series, we observed that shorter periods of both TKI treatment and MR4.5 before TKI discontinuation were significantly associated with increased risk of molecular recurrence, with the best cutoffs for discrimination being at 5 years and 4 years, respectively. Despite that, the predictive value of such clinical factors to accurately identify non-relapsing patients at the individual level was poor. Moreover, when we compared the incidence of molecular relapse in optimal candidates for TKI treatment discontinuation according to the criteria from Hughes and Ross^15^ (58% of patients in our series) with that of the remaining patients, no difference was observed. In line with other authors^16^, we think that the emphasis on defining minimal criteria for discontinuation TKI therapy in clinical practice should not overlook what rate of TFR would be acceptable for each individual patient, depending on the specific TKI-related risks and patient preferences.

In conclusion, the present results contribute to reassure the safety of TKI treatment discontinuation in real-life clinical practice, under close molecular monitoring. Resolution of TKI-related toxicity might translate into a potential health improvement for the patients. Special attention should be paid to the increase in cholesterol levels observed after imatinib discontinuation. Finally, further studies addressing the biological factors underlying the mechanisms of disease control after TKI treatment cessation are warranted.

## Electronic supplementary material

Supplementary Figure Legends

Figure S1 Cumulative incidence of molecular relapse according to the duration of TKI therapy before discontinuation

Figure S2 Cumulative incidence of molecular relapse according to the time in MR4.5 before TKI treatment discontinuation

Figure S3 Cumulative incidence of molecular relapse in optimal candidates for TKI discontinuation in clinical practice (as defined by Hughes & Ross) compared to “less than optimal” candidates
