# Supplementary material for: Feasibility of treatment discontinuation in chronic myeloid leukemia in clinical practice: results from a nationwide series of 236 patients
Source: Blood Cancer J. 2018 Dec 2;8(10):91. doi: 10.1038/s41408-018-0125-0 (PMC6275158; doi:10.1038/s41408-018-0125-0)
Supplement: Supplementary file 1 — Supplementary Figure Legends [file 41408_2018_125_MOESM1_ESM.docx]

**Supplementary Legends**

**Supplemental Figure S1**. Cumulative incidence of molecular relapse according to the duration of TKI therapy before discontinuation.

**Supplemental Figure S2**. Cumulative incidence of molecular relapse according to the time in MR4.5 before TKI treatment discontinuation.

**Supplemental Figure S3**. Cumulative incidence of molecular relapse in optimal candidates for TKI discontinuation in clinical practice (as defined by Hughes & Ross) compared to “less than optimal” candidates.
