# Supplementary figures and images for: Feasibility of treatment discontinuation in chronic myeloid leukemia in clinical practice: results from a nationwide series of 236 patients
Source: Blood Cancer J. 2018 Dec 2;8(10):91. doi: 10.1038/s41408-018-0125-0 (PMC6275158; doi:10.1038/s41408-018-0125-0)

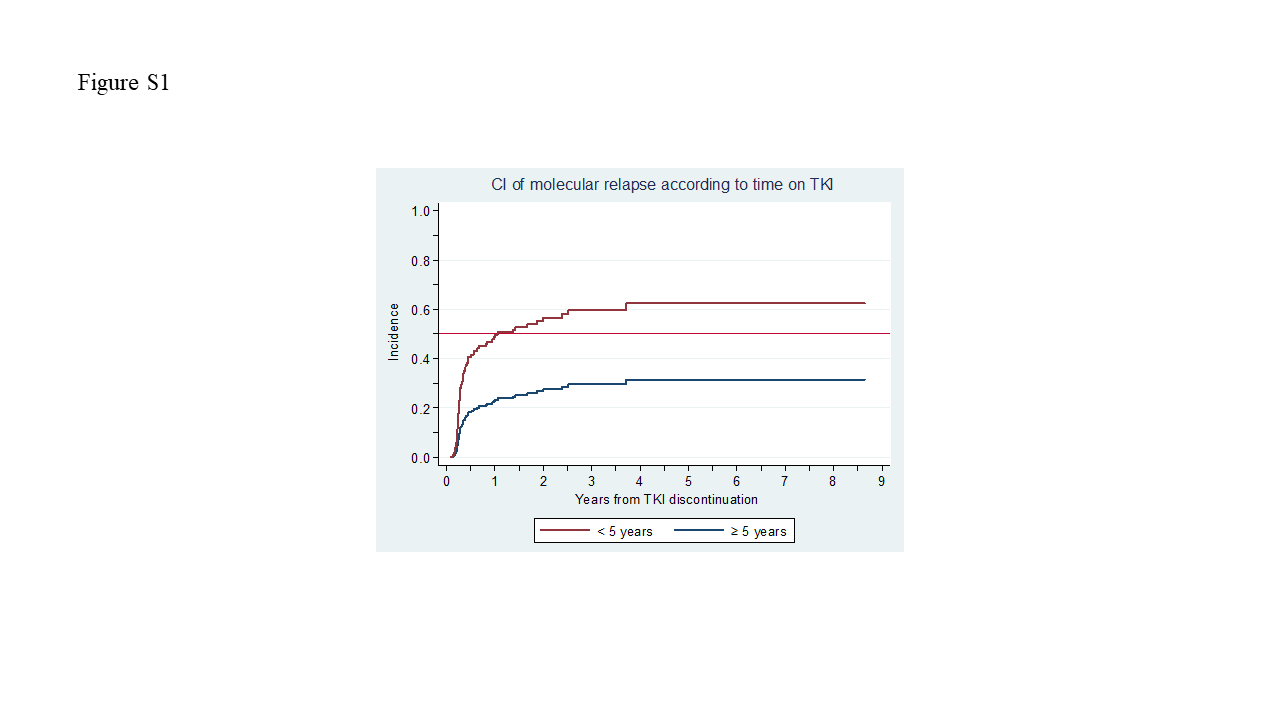

Supplement: Supplementary file 2 — Figure S1 Cumulative incidence of molecular relapse according to the duration of TKI therapy before discontinuation [file 41408_2018_125_MOESM2_ESM.tif]

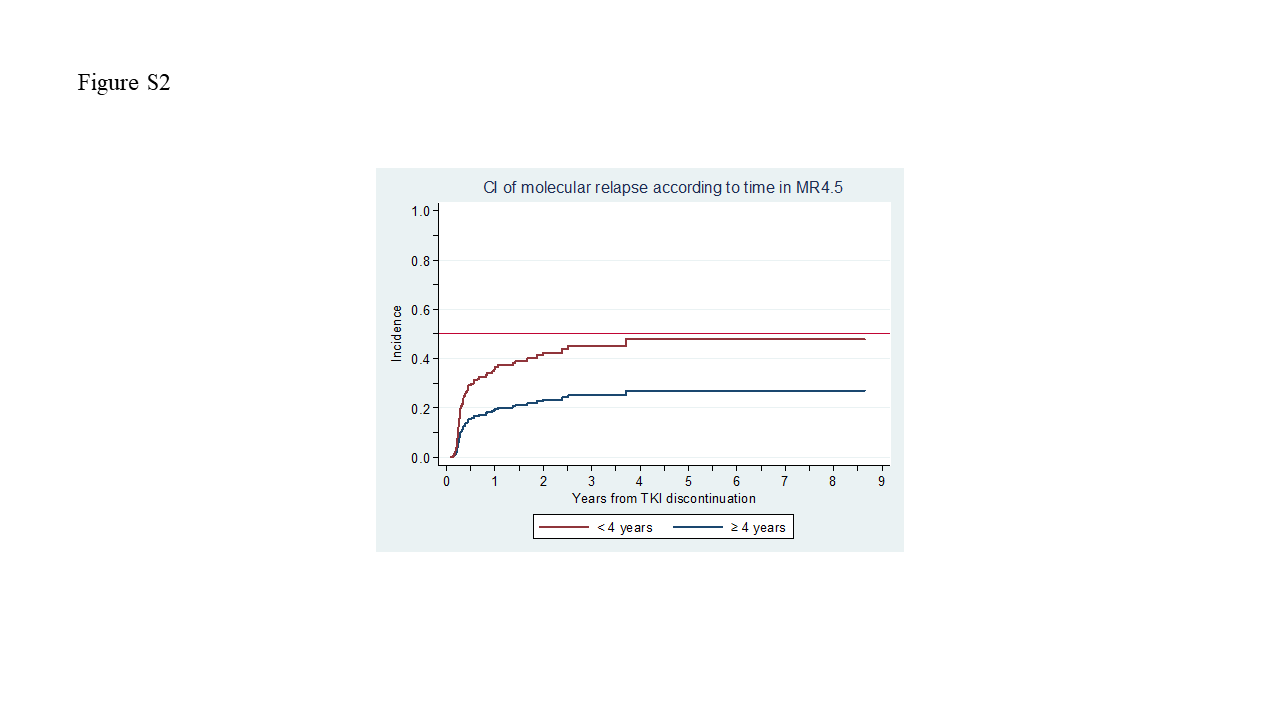

Supplement: Supplementary file 3 — Figure S2 Cumulative incidence of molecular relapse according to the time in MR4.5 before TKI treatment discontinuation [file 41408_2018_125_MOESM3_ESM.tif]

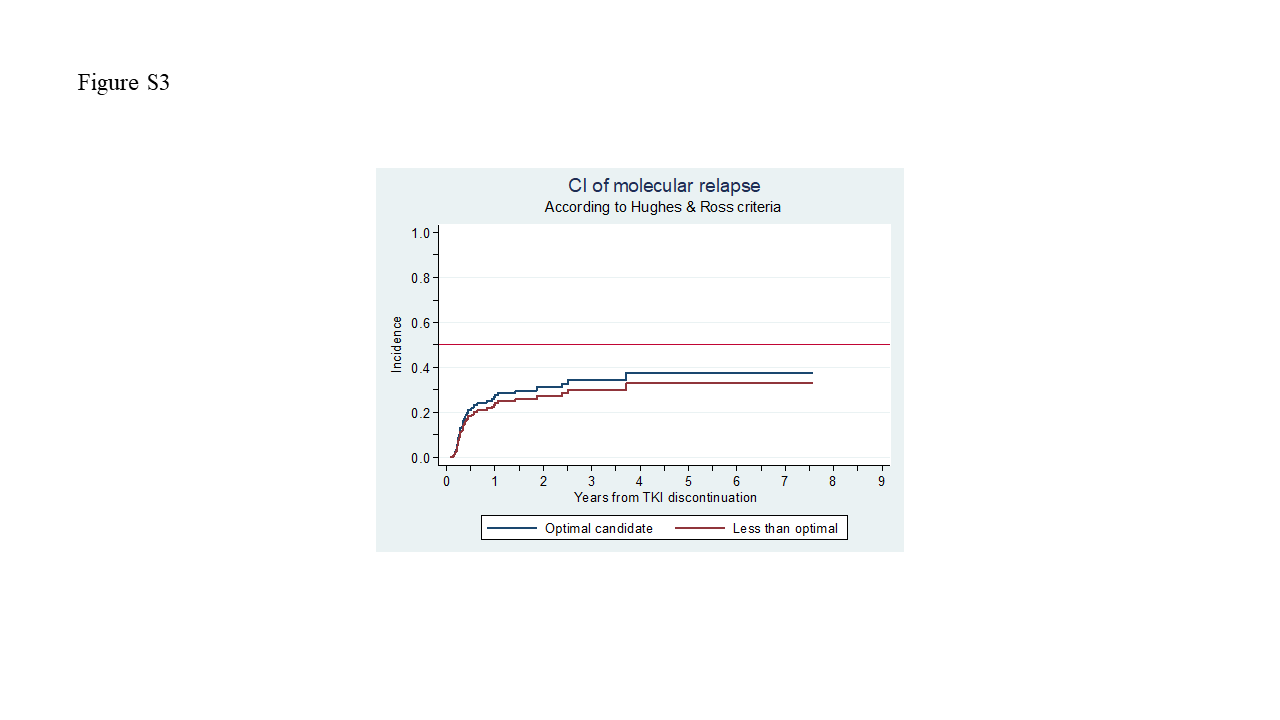

Supplement: Supplementary file 4 — Figure S3 Cumulative incidence of molecular relapse in optimal candidates for TKI discontinuation in clinical practice (as defined by Hughes & Ross) compared to “less than optimal” candidates [file 41408_2018_125_MOESM4_ESM.tif]
